# Damage control strategy in perforated diverticulitis with generalized peritonitis

**DOI:** 10.1186/s12893-021-01130-5

**Published:** 2021-03-16

**Authors:** Maximilian Sohn, Ayman Agha, Igors Iesalnieks, Felix Gundling, Jaroslav Presl, Alfred Hochrein, Dario Tartaglia, Antonio Brillantino, Alexander Perathoner, Johann Pratschke, Felix Aigner, Paul Ritschl

**Affiliations:** 1 Department of General, Abdominal, Endocrine and Minimally Invasive Surgery, Munich Clinic Bogenhausen, Englschalkinger Str. 77, 81925 Munich, Germany; 2 Department of Gastroenterology, Medizinische Klinik II, Sozialstiftung Bamberg, Bamberg Bamberg, Germany; 3 Department of Surgery, Paracelsus Medical University, Salzburg, Austria; 4 OCM Munich, Munich, Germany; 5 Emergency Surgery Unit, Cisanello Hospital, University of Pisa, Pisa, Italy; 6 “A Cardarelli” Hospital, Via A. Cardarelli 9, 80131 Naples, Italy; 7 Department of Abdominal, Transplant and Thoracic Surgery, Medical University Innsbruck, Innsbruck, Austria; 8 Department of Surgery, Barmherzige Brüder Krankenhaus Graz, Graz, Austria; 9 Department of Surgery, Campus Charité Mitte, Campus Virchow-Klinikum, Charité-Universitätsmedizin Berlin, Freie Universität Berlin, Humboldt-Universität Zu Berlin, Berlin Institute of Health, 10178 Berlin, Germany

**Keywords:** Perforated diverticulitis, Peritonitis, Hartmann procedure, Laparoscopic lavage, Primary anastomosis, Damage control surgery

## Abstract

**Background:**

The best treatment for perforated colonic diverticulitis with generalized peritonitis is still under debate. Concurrent strategies are resection with primary anastomosis (PRA) with or without diverting ileostomy (DI), Hartmann’s procedure (HP), laparoscopic lavage (LL) and damage control surgery (DCS). This review intends to systematically analyze the current literature on DCS.

**Methods:**

DCS consists of two stages. Emergency surgery: limited resection of the diseased colon, oral and aboral closure, lavage, vacuum-assisted abdominal closure. Second look surgery after 24–48 h: definite reconstruction with colorectal anastomosis (−/ + DI) or HP after adequate resuscitation. The review was conducted in accordance to the PRISMA-P Statement. PubMed/MEDLINE, Cochrane central register of controlled trials (CENTRAL) and EMBASE were searched using the following term: (Damage control surgery) AND (Diverticulitis OR Diverticulum OR Peritonitis).

**Results:**

Eight retrospective studies including 256 patients met the inclusion criteria. No randomized trial was available. 67% of the included patients had purulent, 30% feculent peritonitis. In 3% Hinchey stage II diverticulitis was found. In 49% the Mannheim peritonitis index (MPI) was greater than 26. Colorectal anastomosis was constructed during the course of the second surgery in 73%. In 15% of the latter DI was applied. The remaining 27% received HP. Postoperative mortality was 9%, morbidity 31% respectively. The anastomotic leak rate was 13%. 55% of patients were discharged without a stoma.

**Conclusion:**

DCS is a safe technique for the treatment of acute perforated diverticulitis with generalized peritonitis, allowing a high rate of colorectal anastomosis and stoma-free hospital discharge in more than half of the patients.

**Supplementary Information:**

The online version contains supplementary material available at 10.1186/s12893-021-01130-5.

## Background

Perforated diverticulitis of the colon is among the most common emergencies in abdominal surgery in industrialized Western countries. To date, the best treatment approach is still a matter of controversy. Between 2010 and 2019, ten relevant international guidelines focusing on that topic were published [1–10]. Concurrent techniques include Hartmann´s procedure, resection with primary anastomosis with or without diverting ileostomy, laparoscopic lavage and a two-stage damage control strategy. Currently, there is no clear consensus among the various national guidelines as to which method should be preferred. Nine of ten of the above-mentioned guidelines were published before 2017 [3–10]. The latest data included in these guidelines are from 2015. Thus, a relevant number of studies on DCS were not included. DCS is suggested only by Sartelli et al. in the World Society of Emergency Surgery guidelines for critical patients to “enhance sepsis control and improve the rate of anastomosis” [3]. Recently updated, the practice parameters of the American Society of Colorectal Surgeons recommends to choose the respective approach depending on patient- and intraoperative characteristics as well as on the treating surgeon’s preference. Apart from that, no clear recommendation for one of the available approaches is given [11]. The new guidelines of the European Society of Coloproctology state that overt perforation shall be treated in accordance to the surgeon’s experience. For perforations with purulent peritonitis in Hinchey stage III, laparoscopic lavage was assessed to be appropriate in selected patients while resection is alternatively recommended. This can be applied establishing a primary anastomosis with or without diverting ileostomy in hemodynamically stable patients. DCS is merely mentioned as an existing strategy [12]. Even the latest guidelines do not frame a “golden standard”. Therefore, the present systematic review was conducted to provide a comprehensive analysis of the current literature on DCS for the treatment of perforated diverticulitis complicated by generalized peritonitis.

## Rationale and objectives

Hypothesis of the presented systematic review is, that the application of the damage control strategy defined above leads to a lower stoma rate than the use of concurrent approaches, without negatively influencing morbidity and mortality.

## Methods

The systematic review is constructed in accordance to the “Preferred Reporting Items for Systematic Reviews and Meta-analyses” (PRISMA) statement as well as to the suggestions of the Cochrane Handbook for systematic reviews [13, 14]. The PRISMA and AMSTAR2 checklist are available in the Additional files 1, 2 [13, 15]. The review methods are based on a protocol established prior to the start of the systematic search.

## Eligibility criteria

### Study designs and inclusion criteria

The PICOS of this systematic review is depicted in Table 1. Randomized and non-randomized studies published in English-speaking, peer-reviewed journals were eligible for the systematic review. No restrictions were made in regard to the date of publication. Congress articles, articles in other languages than English and German, case reports and previous systematic reviews with or without meta-analysis were excluded consequently, but screened for additional sources. If more than one study per institution or collaborative data from two institutions were identified, the authors were asked to separate potential overlaps to reduce the risk for doubled inclusion of patients. Table 2 depicts inclusion as well as exclusion parameters.

**Table 1:** PICOS-Question

**P**	**P**atient, **P**opulation, **P**roblem	Patients with perforated diverticulitis and generalized purulent or feaculent peritonitis. No restrictions on comorbidities, age groups or sex
I	**I**ntervention, Prognostic Factor, or Exposure	Patients who underwent a two staged damage control strategy
C	**C**omparison or Intervention (if appropriate)	Patients who were treated by a concurrent approach: primary anastomosis with or without diverting ileostomy, Hartmann´s procedure
O	**O**utcome you would like to measure or achieve	Stoma rate at discharge, anastomotic leak rate, morbidity, mortality, unplanned revision laparotomy, rate of fascia closure
S	**S**tudy types	Randomized, non-randomized, prospective, retrospective

**Table 2:** Inclusion- and exclusion parameters

Inclusion parameters	Exclusion parameters
*Journal type*	
Peer-reviewed	Non-peer reviewed
*Study type*	
Randomized	Congress articles
Non randomized	Case reports
Prospective	Case series (< 5 patients)
Retrospective	Non-systematic reviews
	Systematic reviews with or without meta-analysis
	Redundant studies from one center if double inclusion of patients could not ruled out definitely
*Language*	
English German	Other
*Diagnosis*	
Perforated diverticulitis of the left colon with generalized purulent or fecal peritonitis	Studies on sealed perforation and/or localized peritonitis
*Therapy*	
Damage control surgery	Primary anastomosis with or without diverting ileostomy
	Hartmann´s procedure
	Laparoscopic lavage

## Definition

DCS is defined as a two-stage procedure: first, a limited resection of the diseased colonic segment with oral and aboral blind closure, lavage, and temporary vacuum-assisted abdominal closure is performed during emergency surgery. Vacuum assistance was the only technique used for temporary abdominal closure within the analyzed cohort. 24–48 h later, the patient undergoes a second-look operation and definite reconstruction with colorectal anastomosis (−/ + diverting ileostomy) or end-colostomy (secondary HP) under optimized conditions after adequate resuscitation. Due to a lack of precise grading systems for the evaluation of the remission of peritonitis, it was coded binary as complete macroscopic clearance of the abdominal cavity without remaining pus or feces. Prior, this could be shown to have a significant impact on the clinical outcome of affected patients [24].

## Data sources and search strategy

**Fig. 1:**
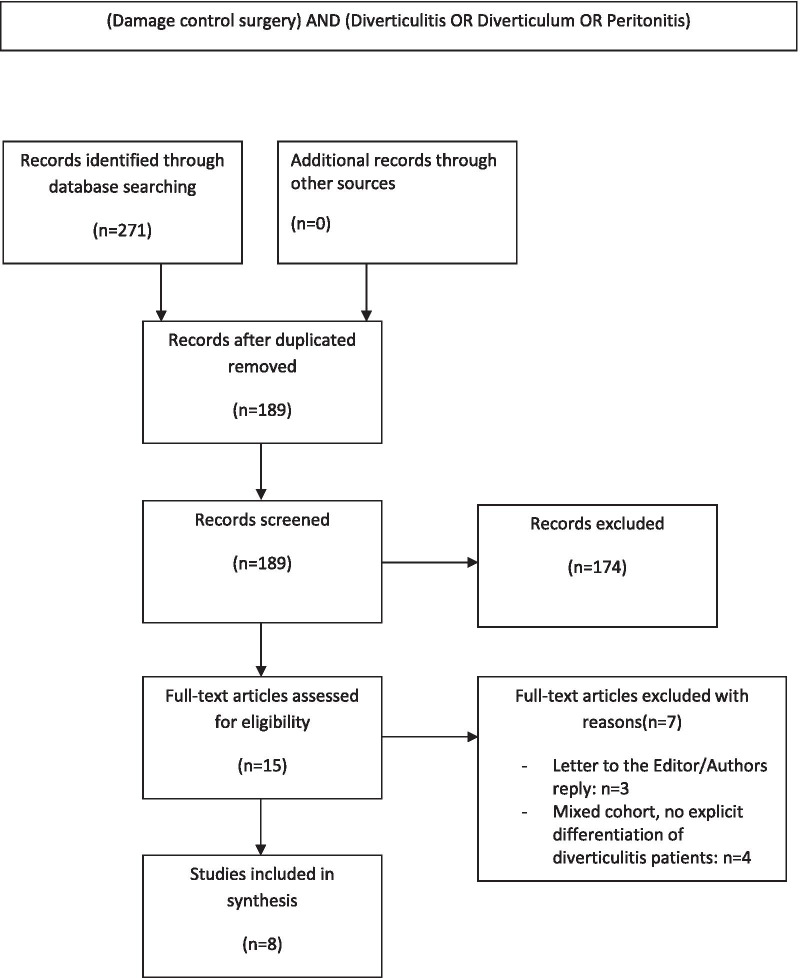
PRISMA flow chart

PubMed/MEDLINE, Cochrane central register of controlled trials (CENTRAL) and EMBASE were systematically screened. Therefore, the below mentioned search-term was developed: (Damage control surgery) AND (Diverticulitis OR Diverticulum OR Peritonitis). To extend potential hits, the “related articles” function of PubMed was used. Additionally, all references of selected articles were screened by hand-search for additional publications matching to the inclusion criteria. As additional sources, the Clinical Trials Registry Platform Search Portal and ClinicalTrials.gov were screened for ongoing or recently completed studies. To avoid unnecessary double-publication, the PROSPERO-Database and the Review Registry Database for systematic reviews and metaanalyses were checked for similar systematic reviews currently underway or finalized. All abstracts and full-text articles were screened for the below mentioned inclusion criteria by two independent researchers. The search was completed June 30^th^, 2020. The search strategy is depicted in Fig. 1.

## Study records

### Data management

All articles identified by database search were stored in a Microsoft Excel database. In case of exclusion of a publication, reasons were attributed. After completed selection of all full-text articles, data were extracted using standardized data extraction forms by MS and PR.

### Data selection process

All reports were independently screened for predefined data items by two authors (MS, PR) through each phase of the review. If any inconsistency occurred concerning in- or exclusion of a study, data was presented to a third independent researcher (DW) to draw a final decision. In cases with incomplete data, the authors of the original studies were contacted to provide lacking information.

## Data items

The following parameters were assessed: ASA, Hinchey stage (presence of purulent or feculent peritonitis), Mannheim peritonitis index (MPI) [16–18] at initial surgery, formation of colorectal anastomosis at second surgery (2ndS), diverting ileostomy at 2ndS, HP at 2ndS, remission of peritonitis at 2ndS, rate of primary wound closure at 2ndS, overall morbidity, rate of postoperative wound dehiscence, anastomotic leakage, need for revision laparotomy, mortality, stoma rate at the time of hospital discharge and stoma rate at the time of the last follow-up.

## Outcomes and prioritization

Primary outcome parameter

- Stoma rate at discharge from the hospital

Secondary outcome parameter

- Rate of colorectal anastomosis at second surgery
- Anastomotic leak rate
- Morbidity
- Mortality
- Rate of fascia closure

## Quality assessment and risk of publication bias

According to the recommendations of the Cochrane network, the Risk of Bias in Non-randomized Studies—of Interventions tool (ROBINS-I) [19] was used to assess methodological quality of included studies. Thereby, studies were screened and judged for a low, moderate, serious or critical risk of confounding bias, selection bias or bias occurring due to different definition or explanation of interventions, missing data, measurement of outcome or reporting results and an overall estimated risk of bias is estimated [19]. In this context, quality assessment reflects how well the identified study is associated with the primary endpoint of this systematic review regardless of the primal objective of the included study itself Table 3.

**Table 3:** ROBINS-I (Risk of bias judgements in non-randomized studies of interventions)

Author	Year	Confoun-ding	Selection of partici-pants	Classifi-cation of Intervent-ions	Deviation from intended Intervent-ions	Missing Data	Measurement of outcomes	Selection of reported Results	Overall
Perathoner [21]	2010	Serious	Moderate	Low	Low	Low	Low	Moderate	Serious
Kafka-Ritsch [20]	2012	Serious	Moderate	Low	Low	Low	Low	Moderate	Serious
Sohn [22]	2016	Serious	Moderate	Low	Low	Low	Low	Moderate	Serious
Sohn [23]	2018	Moderate	Moderate	Low	Low	Low	Low	Moderate	Moderate
Sohn [24]	2018	Serious	Moderate	Low	Low	Low	Low	Moderate	Serious
Gasser [25]	2018	Serious	Moderate	Low	Low	Low	Low	Moderate	Serious
Tartaglia [26]	2019	Moderate	Moderate	Low	Low	Low	Low	Moderate	Moderate
Brillantino [27]	2019	Serious	Moderate	Low	Low	Low	Low	Moderate	Serious

Low: comparable to a well-performed randomized trial

Moderate: sound for a non-randomized study, not comparable to a randomized trial

Serious: presence of important problems; critical: too problematic to provide any useful evidence on the effects of intervention

Overall risk of bias equal to the most severe level of bias found in any domain

## Data synthesis and statistical methods

A narrative synthesis of the results was performed since meta-analysis was not reasonably possible due to inhomogeneous inclusion criteria and follow-up workflow/follow-up examinations as well as partly lacking control groups. After a thorough analysis of the raw data from the original studies by respective authors or coauthors, a relevant overlap of cohorts was found in all centers with more than one publication on DCS. In a next step multiply analyzed patients were excluded. The review authors decided to report on the adjusted data from the different study groups and not on those given in the original articles. Therefore, data from five study groups rather than eight original studies are presented in Table 4 and in the results section. Results are reported in differences in mean.

**Table 4:** Separate results of included study groups

Variable	SG 1	SG 2	SG 3	SG 4	SG 5
Multiple publication	Yes	Yes	No	No	No
	2010	2016			
	2012	2018			
		2018			
Population	Perforated diverticulitis of the left colon with generalized peritonitis	Perforated diverticulitis of the left colon with generalized peritonitis	Perforated diverticulitis of the left colon with generalized peritonitis	Perforated diverticulitis of the left colon with generalized peritonitis	Perforated diverticulitis of the left colon with generalized peritonitis
Intervention	DCS	DCS	ABTheraTM	DCS	DCS
Comparator	2010: PRA, HP 2012: no	2016: PRA, HP 2018: No 2018:No	SuprasorbVR	No	No
Main outcome	2010: not specified	2016: Not specified	Not specified *Comment* Comparision of different systems for vacuum therapy in DCS patients	Mortality, Morbidity, Rate of intestinal anastomoses at 2nd look surgery	Not specified
	2012: not specified	2018: Stoma rate at discharge			
		2018: Prognostic impact of ongoing peritonitis at 2nd look surgery			
Design	POS	RS	RS	RS	RS
Follow-up	Length not specified	Length not specified	No	47 month (mean)	No
n	51	74	67	34	30
ASA > 2, n (%)	51 (100)	58 (78)	48 (72)	22 (65)	30 (100)
Age	69 (28–87) (median)	67 (30–92) (median)	67 (43–86) (median)	66.9 (mean)	68.5 (35–84) (median)
Female:male (%)	55:45	54:46	78:22	56:44	60:40
Sepsis/septic shock, n (%)	16 (31)	16 (22)	34 (51)	34 (100)	8 (27)
Hinchey III, n (%)	40 (78)	60 (81)	41 (61)	13 (38)	17 (57)
Hinchey IV, n (%)	11 (22)	14 (19)	18 (27)	21 (62)	13 (43)
MPI median	26 (12–39)	22.4 (6–42)	22 (0–39)	25.12	26.2 (12–40)
MPI ≥ 26, n (%)	32 (63)	29 (39)	24 (36)	23 (68)	18 (60)
Colorectal anastomosis at 2nd look operation, n (%)	38 of 50 (74)	62 (84)	37 of 65 (57)	24 (71)	24 (80)
Diverting Ileostomy at 2nd look, n (%)	4 of 50 (8)	25 (34)	6 of 65 (9)	3 (9)	0 (0)
End colostomy at 2nd look, n (%)	12 of 50 (24)	12 (16)	28 of 65 (43)	10 (29)	6 (20)
Peritonitis remission at 2nd look, n (%)	28 of 50 (56)	41 (55)	46 of 65 (71)	27 (79)	24 (80)
Fascia closure at 2nd look, n (%)	50 of 50 (100)	74 (100)	46 of 65 (71)	34 (100)	30 (100)
Surgical Morbidity, n (%)	19 (37)	26 (35)	14 (21)	14 (41)	7 (23)
Anastomotic leak (AL), n (%)	5 of 38 (13)	8 of 62 (13)	9 of 37 (24)	1 of 24 (4)	1 of 24 (4)
Revision laparotomy, n (%)	4 (8)	11 (15)*	19 (28)	3 (9)	0 (0)
Fascia dehisience, n (%)	1 of 50 (2)	5 (7)	5 of 65 (7)	2 (6)	0 (0)
Mortality (30 d), n (%)	5 (10)	5 (7)	9 (13)	4 (12)	1 (3)
Stomarate at discharge, n (%)	16 of 50 (32)	43 (58)	34 of 65 (52)	14 (41)**	6 (20)

SG 1: Perathoner/Kafka-Ritsch et al., University Hospital Innsbruck, Austria; SG 2: Sohn et al., DCS Study Group Munich/Berlin, Germany; SG 3: Gasser et al., University Hospitals Innsbruck/Salzburg, Austria; SG 4: Tartaglia et al., New Santa Chiara Hospital, University of Pisa, Italy; SG 5: Brillantino et al., “A Cardarelli” Hospital, Naples, Italy

*HP*  Hartmann’s procedure, *PRA*  primary anastomosis, *RS* retrospective, *POS*  prospective observational study, *DCS*  damage control surgery

*At revision laparotomy three patients received additional loop ileostomy and three other patients received end colostomy for anastomotic complication

**At revision laparotomy, one patient received end colostomy

## Results

Overall, six retrospective cohort studies and two prospective observational studies from five centers, referring to five different study cohorts, met the inclusion criteria [20–27] (Fig. 1). There were no prospectively randomized trials. At CENTRAL and ClinicalTrials.gov one prospectively randomized trial was indicated. Recruiting status was set to “completed” in July 2019. Since then, no updates were made. This trial is limited by a low number of participants (n = 22). No funding or competing interests were identified in association to any of the included studies. The overall study population consisted of 256 patients (Table 4). Thereof, 58% of patients were female. Median age was available for four study groups, ranging from 67 to 69 years. Tartaglia et al. calculated an age mean of 66.9 years. In total, 67% presented with purulent peritonitis, 30% presented with feculent peritonitis, and 3% were diagnosed with Hinchey stage II diverticulitis. The median Mannheim peritonitis index (MPI) was 26, 22.4, 22, 25.12, and 26.2 in the study group (SG) 1–5, respectively. In 126 patients (49%), the MPI was greater than 26. At the second surgery, colorectal anastomosis was constructed in 185 patients (73%); in 38 of the latter, a diverting ileostomy was formed (15%). An end-colostomy (secondary HP) was performed in 68 patients (27%). In 66% of patients, no macroscopic signs of persistent peritonitis could be found at the second surgery. Complete fascia closure was achieved in 234 patients (92%) at the second surgery. The overall postoperative morbidity was 31%. Anastomotic leaks occurred in 24 patients (13%) who underwent colorectal anastomosis at the second surgery. An unplanned revision laparotomy was necessary in 37 patients (14%), wherein 13 patients (5%) suffered from wound dehiscence. The stoma rate at the time of hospital discharge was 45% (n = 113). Postoperative mortality was 9% (n = 24) (Table 5). The level of association between the identified studies and the primary endpoint of this systematic review was assessed using the ROBINS-I tool [19]. Therein six of eight studies are estimated to have a serious risk of bias while two more studies were classified with “moderate risk”. Highest scores were reached by the studies of Sohn et al. from 2018 and Tartaglia from 2019 because an adjustment for confounders was made by logistic regression analysis. A major flaw of all analyses was the fact, that decision for stoma-formation at the second surgical step was made on a more or less individual base according to the treating surgeons appraisal. Moreover, only Perathoner (2010) and Sohn (2016) compared the two-staged DCS approach with a conventional “one-step” surgery (Table 3).

**Table 5:** Cumulative perioperative findings

Variable	Results, n (%)
*Findings at emergency surgery*	
ASA > 2	209 (82)
Sepsis/septic shock	108 (42)
Hinchey III	171 (67)
Hinchey IV	77 (30)
MPI > 26	126 (49)
*Characteristics at second surgery*	
Colorectal anastomosis	185 of 253 (73)
Diverting ileostomy	38 of 253 (15)
End colostomy	68 of 253 (27)
Macroscopic remission of peritonitis	166 of 253 (66)
Rate of fascia closure	234 of 253 (92)
*Postoperative characteristics*	
Surgical morbidity	80 (31)
Anastomotic leak	24 of 185 (13)
Revision laparotomy	37 of 253 (15)
Additional ileostomy at revision laparotomy	3 of 253 (1)
Additional colostomy at revision laparotomy	4 of 253 (2)
Fascia dehicience	13 of 253 (5)
Stoma rate at discharge	113 of 253 (45)
Mortality	24 of 256 (9)

## Discussion

**Fig. 2:**
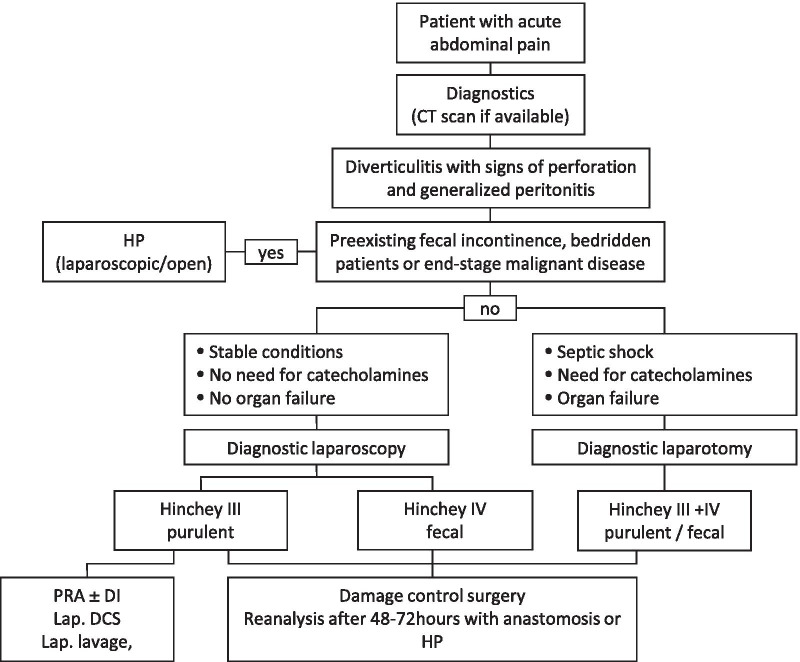
Diagnostic and therapeutic workup

Although perforated colonic diverticulitis complicated by generalized peritonitis constitutes a frequent abdominal emergency, an internationally accepted treatment approach has yet to be established. In descending order of importance, the aim of every therapy for perforated diverticulitis should be low mortality, low morbidity, low stoma rate, ease of performance and practicability, and low cost. According to current evidence, resection with primary anastomosis and diverting ileostomy seems to be an appropriate approach for therapy in most patients with perforated diverticulitis complicated by purulent and fecal peritonitis. Meanwhile, four prospectively randomized trials demonstrated this approach to be quite safe and feasible in most cases [28–31]. The authors concluded that PRA is preferable to HP in terms of significantly better stoma-free survival, while morbidity and mortality were found to be without significant differences in the short term. Altogether, the results of randomized trials showed that HP was associated with a higher rate of definite stoma, a longer time to stoma closure and a higher rate of overall complications when the reversal procedure was included. In the evaluation of LL for perforated diverticulitis and purulent (but not feculent) peritonitis, the results of three randomized trials were available [32–34]. While Angenete et al. found no inferiority of LL in comparison to HP in the short-term and the two-year follow-up [32, 35] the authors of the SCANDIV Trial [33] and of the LOLA arm of the LADIES [34] trial did not recommend routine use of LL due to an increased event rate in the LL group. Importantly, LL was not applied to any patient with fecal peritonitis in any of the mentioned studies. Actually, fecal peritonitis was an exclusion criterion in all mentioned studies. According to a meta-analysis of those three randomized trials, Acuna et al. stated in 2017 that LL was associated with a higher risk of postoperative major complications, even though the early reoperation rate and mortality were equal. In the same meta-analysis, a comparison between PRA and HP showed an increased rate of restored bowel continuity after PRA and a higher risk of major complications after stoma reversal in the HP group. Therefore, PRA was highlighted as the preferable approach by the authors [36]. However, this approach was associated with a diverting ileostomy rate of 100% in all studies included in that particular meta-analysis. Moreover, PRA can be a technically challenging procedure in the presence of severe intra-abdominal inflammation. Since emergency surgery is often performed outside of the daytime schedule, routine implementation of PRA may be difficult. According to our experience and contrary to the current literature, surgeons often tend to avoid PRA in ongoing peritonitis in favor of HP when facing surgical “real life” conditions. Interestingly, even in one prospectively randomized multicenter study that compared PRA with diverting ileostomy and HP, 10% of randomized PRA patients finally received HP due to unexplained surgeon choices [37]. Furthermore, a national retrospective cohort study by Cauley et al., including data collected between 1998 and 2011 [38], showed that the overall use of PRA in the USA was very low (3.9% vs. 96.1% of end colostomies). Using weighted estimates, Cauley and coworkers calculated an end colostomy rate of more than 90% for 2011. Similarly, Roig et al. found a prevalence of HP in 72.2% of cases within a retrospective series of 358 patients with perforated diverticulitis and peritonitis [39]. Thus, it was the commonest approach. PRA was performed in only 17.9% of that cohort. A lack of experienced colorectal specialists during nighttime shifts further aggravates the present problems. Thus, alternate strategies to PRA are needed for the treatment of patients with perforated diverticular disease, especially in cases of feculent peritonitis. DCS is applicable even in the latter case, and colorectal anastomosis can be constructed in > 75% of all patients, as shown in our analysis. The rate of end colostomy (22%) as well as the overall stoma rate (including diverting ileostomy) at discharge (46%) is relatively low. The first surgical step is easily applicable even in the absence of a colorectal specialist, while the decision for definite reconstruction is postponed to a situation with optimized conditions and the support of a colorectal surgeon. To date, no validated parameters exist for the decision of whether a diverting ileostomy (DI) should be added to colorectal anastomosis during the second surgery. This aspect is supported by the relevantly different rate of DI established in the analyzed studies. We found a range of 0 to 34% for DI, while no clear association between the DI rate and anastomotic leakages could be shown. In one study, the presence of macroscopically persisting peritonitis at the second surgery was associated with increased overall morbidity. Enterococcal (81% vs. 44%, p = 0.005) and fungal infection (100% vs. 49%, p = 0.007) during the emergency laparotomy led to a significantly higher rate of ongoing peritonitis at the second surgery [24]. Evidence of Enterococcus spp. was associated with a higher risk of anastomotic leak (29% vs. 6%, p = 0.042). Thus, a diverting ileostomy should be discussed in cases of ongoing peritonitis, especially when they are caused by enterococcal infection. This question needs to be addressed in future research. Noticeably, end colostomy was applied in 16–43% of cases at the second surgery in the present analysis. This range must be critically challenged because precise initial patient selection might have been expedient for avoiding unnecessary operations. Nevertheless, all concurrent techniques should be weighed carefully in each patient (Table 6). If patients’ conditions and technical requirements are optimal, a primary (laparoscopic) PRA is worth considering, possibly without a diverting ileostomy. In the case of relevant immunosuppression, preexisting fecal incontinence, bedridden patients or end-stage malignant disease, primary HP may be suggested to avoid unnecessary reoperations. In all other conditions, DCS is a safe and reliable option to choose. DCS use should not generally be limited to fecal peritonitis but can be chosen in all conditions where an HP could be avoided. As a future evolution of this technique, laparoscopic DCS is currently under evaluation as a potential development of the technique. A therapeutic algorithm as suggested by the authors of this review is depicted in Fig. 2.

**Table 6:** Characteristics of available strategies for the treatment of perforated diverticulitis with generalized peritonitis

	Damage control surgery	Laparoscopic lavage	Primary anastomosis	Hartmann‘s procedure
Use for Hinchey stage	III + IV	III	III + IV	III + IV
Technical requirements	Low	Medium	High	medium
Risk for Stoma	~ 50%	Low	100%*	100%
Advantages	Rapid and easy focus control, low stoma rate	Minimally invasive treatment, no resection, no stoma	Focus control, definite treatment within one surgical procedure	Focus control, no anastomosis
Disadvantages	Two surgical procedures	Relevant rate of adverse events	Technical challenging, high stoma rate	Low reversal rate, low quality of life

*Following evidence of currently available randomized controlled trials

## Conclusion

DCS fulfills all requirements for emergency surgery: short duration, limited surgical trauma, effective elimination of the septic focus and low technical demands. Therefore, it may be considered a potential treatment option for perforated diverticulitis with both generalized purulent and feculent peritonitis. DCS leads to a low stoma rate compared to other resectional procedures.

The presented study is confined by certain limitations. Data arose of non-randomized studies only. No randomized trials were identified in the study period. Thereby, publication- and selection bias could not be ruled out definitely.

## Supplementary Information

**Additional file 1:** Prisma checklist.

**Additional file 2:** AMSTAR2 checklist.

## Data Availability

Raw-data are available on reasonable request from the first author.

## Abbreviations

PRA: Primary anastomosis
DI: Diverting ileostomy
HP: Hartmann’s procedure
LL: Laparoscopic lavage
DCS: Damage control surgery
PRISMA: Preferred reporting items for systematic reviews and metaanalyses
MPI: Mannheim peritonitis index
PICOS: (P) patient/population/problem; (I) intervention/prognostic factor/exposure; (C) comparison or intervention (if appropriate); (O) outcome; (S) study types
CENTRAL: Cochrane central register of controlled trials
ASA: American Society of Anaesthesiologists Classification
2ndS: Second surgery
ROBINS-I: Risk of bias in non randomized studies-of interventions tool
